# Epidemiological profile of arboviruses in two different scenarios: dengue circulation *vs.* dengue, chikungunya and Zika co-circulation

**DOI:** 10.1186/s12879-023-08139-6

**Published:** 2023-03-22

**Authors:** Pablo Cantalice Santos Farias, André Filipe Pastor, Juliana Prado Gonçales, Iasmyn Dayanne Santos do Nascimento, Ellida Suellen de Souza Ferraz, Thaísa Regina Rocha Lopes, Rodrigo Feliciano do Carmo, Maria Rosângela Cunha Duarte Côelho, José Valter Joaquim Silva Júnior

**Affiliations:** 1 Department of Genetics, Federal University of Pernambuco, Recife, Pernambuco Brazil; 2 Federal Institute of Education, Science and Technology of Sertão Pernambucano, Floresta, Pernambuco Brazil; 3 Department of Microbiology, Icahn School of Medicine at Mount Sinai, New York, USA; 4 Virology Sector, Keizo Asami Institute, Federal University of Pernambuco, Recife, Pernambuco Brazil; 5 SER Educational Group, Recife, Pernambuco Brazil; 6 Laboratory of Immunogenetics, Department of Immunology, Aggeu Magalhães Institute, Recife, Pernambuco Brazil; 7 Virology Sector, Department of Preventive Veterinary Medicine, Federal University of Santa Maria, Av. Roraima, Camobi, Santa Maria, Rio Grande do Sul 97105-900 Brazil; 8 Collegiate of Pharmaceutical Sciences, Federal University of Vale do São Francisco, Petrolina, Pernambuco Brazil; 9 Department of Clinical Analysis, Health Sciences Center, Federal University of Santa Maria, Santa Maria, Rio Grande do Sul Brazil

**Keywords:** Arbovirus co-circulation, Female, Brown, Public Health, Brazil

## Abstract

**Background:**

The severity and distribution of dengue virus (DENV) infections have been attributed to a complex interaction among viral, host and environmental factors. Herein, we investigated the influence of chikungunya (CHIKV) and Zika (ZIKV) viruses on the epidemiological profile of dengue cases, using Recife, Pernambuco state, Brazil, as a study model. In addition, we described and compared the epidemiological profile related to each arbovirus (DENV *vs*. CHIKV *vs*. ZIKV).

**Methods:**

All cases of dengue, chikungunya and Zika reported to the Pernambuco Health Department in 2011–2013 (DENV circulation) and 2016–2018 (DENV, CHIKV and ZIKV co-circulation) were included in our study. The cases were classified by sex, age and race/color and their distribution was analyzed by the χ^2^ test. Furthermore, the data were also analyzed for co-infections. Temperature, humidity and rainfall data were analyzed using one-way ANOVA and paired t-test.

**Results:**

During 2011–2013, 15,315 dengue cases were diagnosed, most of them female, brown and 20–29 age group. Between 2016 and 2018, 15,870 dengue cases were described, which presented the same profile described above. In the two triennia, the female/male dengue ratio fluctuated significantly, ranging from 1.07 to 1.52. Regarding chikungunya, 7076 cases were reported, most of them female and brown. The female/male ratio also fluctuated significantly, ranging from 1.62 to 2.1. Two main age groups were observed in chikungunya: ≤ 19 years (minority of diagnoses) and ≥ 20 years (majority of diagnoses). In the same triennium, 266 Zika cases were reported to the Pernambuco Health Department, mainly in females and in the 0–9 and 20–39 age groups. In general, 119 co-infections were identified: 117 DENV-CHIKV, 1 CHIKV-ZIKV and 1 DENV-CHIKV-ZIKV. Concerning climate data, only the humidity in 2011 was significantly different from the other years.

**Conclusion:**

The epidemiological profile of dengue cases did not change after the introduction of CHIKV and ZIKV. Females were the most diagnosed with dengue, chikungunya or Zika, however we found important differences in the age profile of these arboviruses, which should be considered by public health policies, as well as investigated in future studies of virus-host interaction.

## Highlights

- The introduction of chikungunya and Zika viruses did not change the epidemiological profile of dengue cases in our study population.
- Most cases of dengue, chikungunya and Zika have been described in women.
- Dengue, chikungunya and/or Zika cases had different age profiles.
- The female/male ratio in chikungunya was higher than that observed in dengue cases.
- The epidemiological differences among dengue, chikungunya and Zika must be considered by public health policies.

## Introduction

Dengue virus (DENV) belongs to the family *Flaviviridae*, genus *Flavivirus*, has four serotypes (DENV 1–4) and is one of the most important and widespread arthropod-borne viruses [1, 2]. Over the years, millions of dengue cases have been reported, especially in tropical and subtropical regions. It is estimated that 390 million DENV infections occur annually and 3.9 billion people are at risk [3].

The number of dengue cases and deaths is the result of a complex interaction among viral, host and environmental factors. The introduction of new serotypes and the antibody-dependent enhancement (ADE) phenomenon have been often associated with DENV infections and/or outbreaks [4–10]. Environmental factors, such as temperature, humidity and rainfall, which may influence the population of vector mosquitoes, also contribute to the increase in dengue numbers [11–14]. Moreover, social factors (*e*.*g*., age, sex and race/ethnicity) have also been associated with the risk of DENV infections [15–18].

In addition, several countries and territories have experienced DENV, chikungunya (CHIKV) and Zika (ZIKV) viruses co-circulation [19]. CHIKV and ZIKV are also arboviruses, belonging to the family *Togaviridae* and *Flaviviridae*, respectively [2]. While CHIKV is related to severe joint pain and long-term morbidity [20–22], ZIKV usually causes mild illness, but has also been associated with a congenital syndrome, which may lead to a spectrum of birth defects, including microcephaly [23].

The DENV, CHIKV and ZIKV co-circulation has raised serious issues about potential cross-immunity or ADE between DENV and ZIKV, as well as possible influences on the co-infected vectors' fitness [19, 24–28]. This context has been quite important in Brazil, a country historically endemic for DENV, which had the first autochthonous transmissions of CHIKV and ZIKV described in 2014 and 2015, respectively [29, 30]. Since then, Brazil has reported the co-circulation of these three arboviruses [31], making the country an interesting territory to analyze the interaction among them.

Considering the above, we investigated the potential influence of CHIKV and ZIKV circulation on the epidemiological profile of dengue cases, adopting Recife, Pernambuco state, Brazil, as the study region/model. In addition, we also described chikungunya and Zika cases and compared the epidemiological profiles related to each arbovirus. Overall, the findings reported here provide insights into the dynamics and epidemiology of arboviruses.

## Methodology

### Study design

This work is an observational and analytical cross-sectional study of confirmed cases of dengue, chikungunya and Zika in Recife, which were reported to the Pernambuco Health Department in the 2011–2013 (DENV circulation) and 2016–2018 (DENV, CHIKV and ZIKV co-circulation) triennia.

### Data collection

All confirmed cases of dengue, chikungunya and Zika reported to the Pernambuco Health Department were included in this study. Confirmations were based on laboratory or clinical-epidemiological diagnosis. For each diagnosis, information about sex (male or female), age (0–4, 5–9, 10–14, 15–19, 20–29, 30–39, 40–49, 50–59 or > 60) and race/color (white, black, yellow, brown or indigenous, based on self-identification) was collected. In addition, the data were also analyzed for co-infections. All data were made available by the Pernambuco Health Department (protocol number 2300000157.000776/2020-76).

Temperature and humidity information was collected from the National Institute of Meteorology (*Instituto Nacional de Meteorologia*, INMET) platform [32]. Rainfall data were obtained from the State Agency of Water and Climate (*Agência Pernambucana de Águas e Clima*, APAC) database [33].

### Statistical analyses

Statistical analyses were performed using Epi Info v.7 and Graphpad prism v.5. The distribution of cases by sex, race/color and age group was evaluated by the χ^2^ test. Temperature, humidity and rainfall analyses were performed using the one-way analysis of variance (one-way ANOVA) and paired t-test. A *p-value* < 0.05 and a 95% confidence interval were considered in all analyses.

## Results

### DENV circulation (2011–2013)

**Fig. 1:**
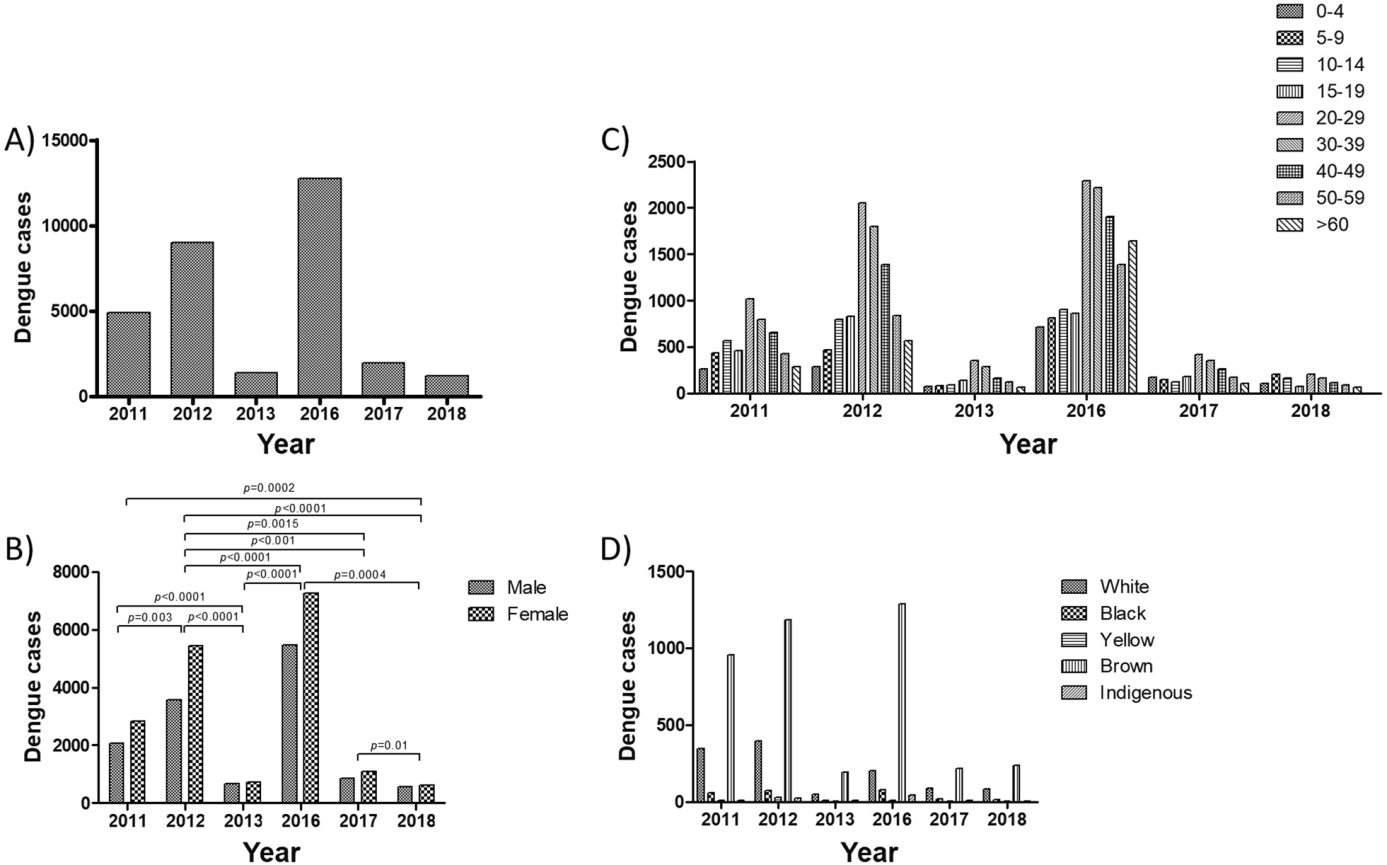
Dengue cases in Recife city, Brazil. Dengue cases were analyzed considering the total number of cases per year (**A**) and their distribution according to sex (**B**), age group (**C**) and race/color (**D**). Cases were reported between 2011 and 2013 (DENV circulation) and between 2016 and 2018 (DENV, CHIKV and ZIKV co-circulation). Data were made available by the Pernambuco Health Department. Statistical analyses were performed using the χ^2^ test. A *p-value* < 0.05 and a 95% confidence interval were considered in all analyses. DENV: dengue virus; CHIKV: chikungunya virus; ZIKV: Zika virus

In 2011, 2012 and 2013, 4915, 9017 and 1383 individuals were confirmed with dengue, respectively, totaling 15,315 cases (Fig. 1A). Females were the most diagnosed over the years: the female/male ratios were 1.37 (2011), 1.52 (2012) and 1.07 (2013) (Fig. 1B). The female/male ratio increased significantly between 2011 and 2012 (*p* = 0.003), decreased significantly between 2011 and 2013 (*p* < 0.0001) and between 2012 and 2013 (*p* < 0.0001) (Fig. 1B).

Regarding age groups, most diagnoses were observed in individuals aged between 20 and 29 years old (Fig. 1C). The frequency of diagnosis in this age group increased during the period: in 2011, 2012 and 2013, individuals aged 20–29 years accounted for 20.7, 22.7 and 25.6%, respectively. On the other hand, individuals in the first years of life, mainly 0–4 years, and over 60 years old corresponded to the least diagnosed groups (Fig. 1C).

Brown was the race/color with the highest number of diagnoses between 2011 and 2013, a prevalence that has also increased over the years: 69.6% (2011), 69.7% (2012) and 74.2% (2013) (Fig. 1D). Race/color information was not collected from 72% (3539/4915), 81.2% (7320/9017), and 81.2% (1123/1383) individuals in 2011, 2012, and 2013, respectively.

### DENV, CHIKV and ZIKV co-circulation (2016–2018)

#### Dengue

In 2016, 2017 and 2018, 12,730, 1,951, and 1,189 individuals were confirmed with dengue, respectively, with a total of 15,870 cases (Fig. 1A). Females had the highest number of diagnoses, with a female/male ratio of 1.33 (2016), 1.30 (2017) and 1.07 (2018) (Fig. 1B). The female/male ratio was significantly lower in 2018 compared to 2016 (*p* = 0.0004) and 2017 (*p* = 0.01) (Fig. 1B).

Regarding age groups, individuals aged 20–29 years were the most diagnosed in 2016 (18%), 2017 (21.6%) and 2018 (17.1%) (Fig. 1C). The distribution of the other age groups was similar to that observed in the first triennium, except for 2016, in which patients over 60 years old were the fourth largest group (Fig. 1C). Brown race/color was the most diagnosed, corresponding to 79.4, 65.4 and 69.8% in 2016, 2017 and 2018, respectively (Fig. 1D). Race/color information was not collected from 87.2% (11,105/12,730), 83.1% (1621/1951), and 71.8% (854/1189) individuals in 2016, 2017 and 2018, respectively.

### Chikungunya

**Fig. 2:**
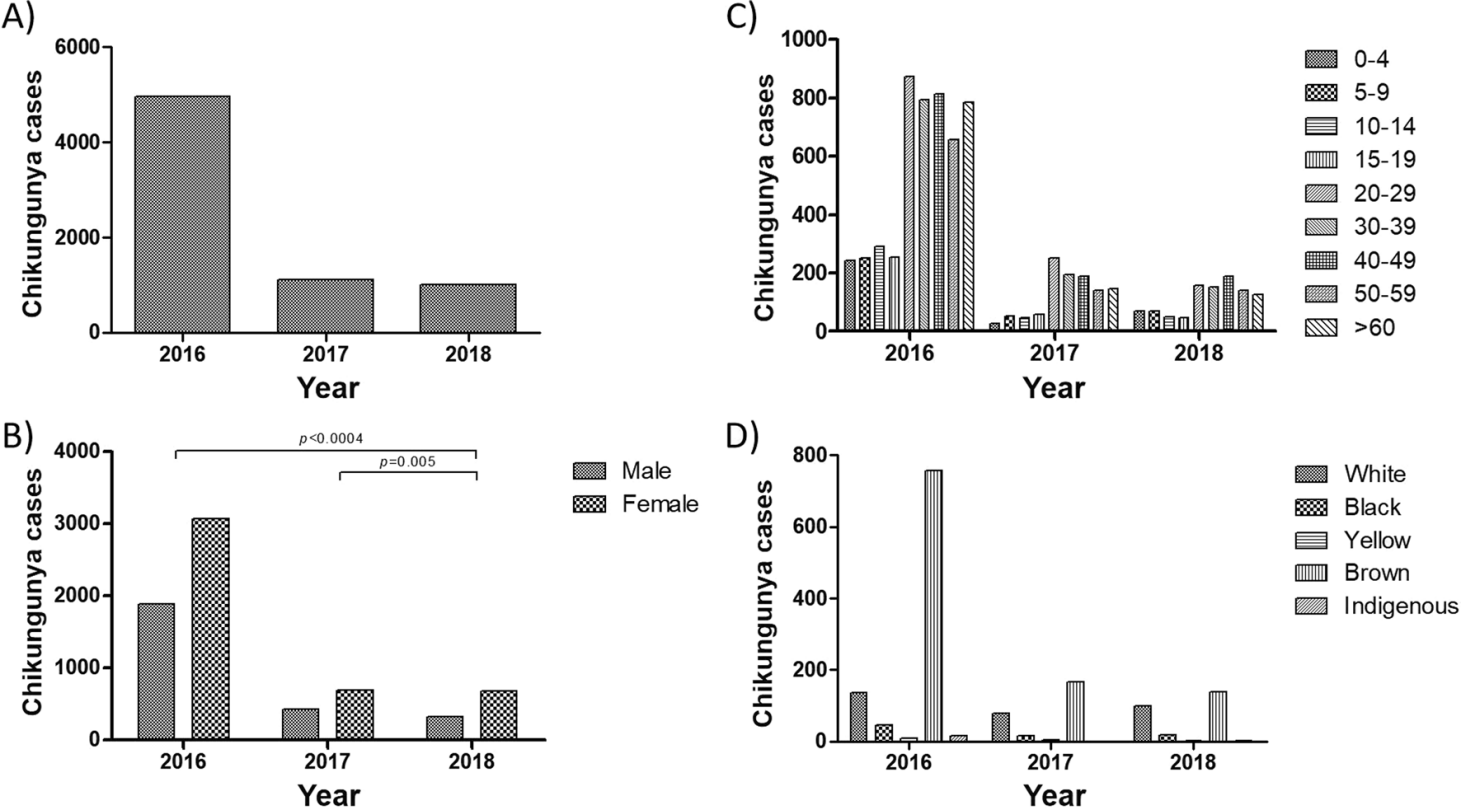
Chikungunya cases in Recife city, Brazil. Chikungunya cases were analyzed considering the total number of cases per year (**A**), sex (**B**), age group (**C**) and race/color (**D**). Statistical analyses were performed using the χ^2^ test (*p-value* < 0.05 and 95% confidence interval). All data were made available by the Pernambuco Health Department

In 2016, 2017 and 2018, 4963, 1108, and 1005 individuals were diagnosed with chikungunya, respectively (Fig. 2A), totaling 7076 cases, reported mainly in female: the female/male ratio was 1.62 (2016), 1.62 (2017) and 2.1 (2018) (Fig. 2B). The increase between 2016 and 2018, as well as between 2017 and 2018, were significant (*p* < 0.0004 and *p* = 0.005, respectively) (Fig. 2B).

The 20–29 age group received the most diagnoses in 2016 (17.6%) and 2017 (22.6%); in 2018, individuals aged 40–49 years were the most diagnosed (18.8%) (Fig. 2C). The distribution of diagnosis among the other age groups was similar among years (Fig. 2C). Brown race/color was the group with the most chikungunya diagnosis: 78.5% (2016), 62.9% (2017) and 52.9% (2018) (Fig. 2D). Race/color information was not collected from 80.6% (3,998/4,963), 76.2% (844/1,108) and 74% (744/1,005) individuals in 2016, 2017 and 2018, respectively.

### Zika

**Fig. 3:**
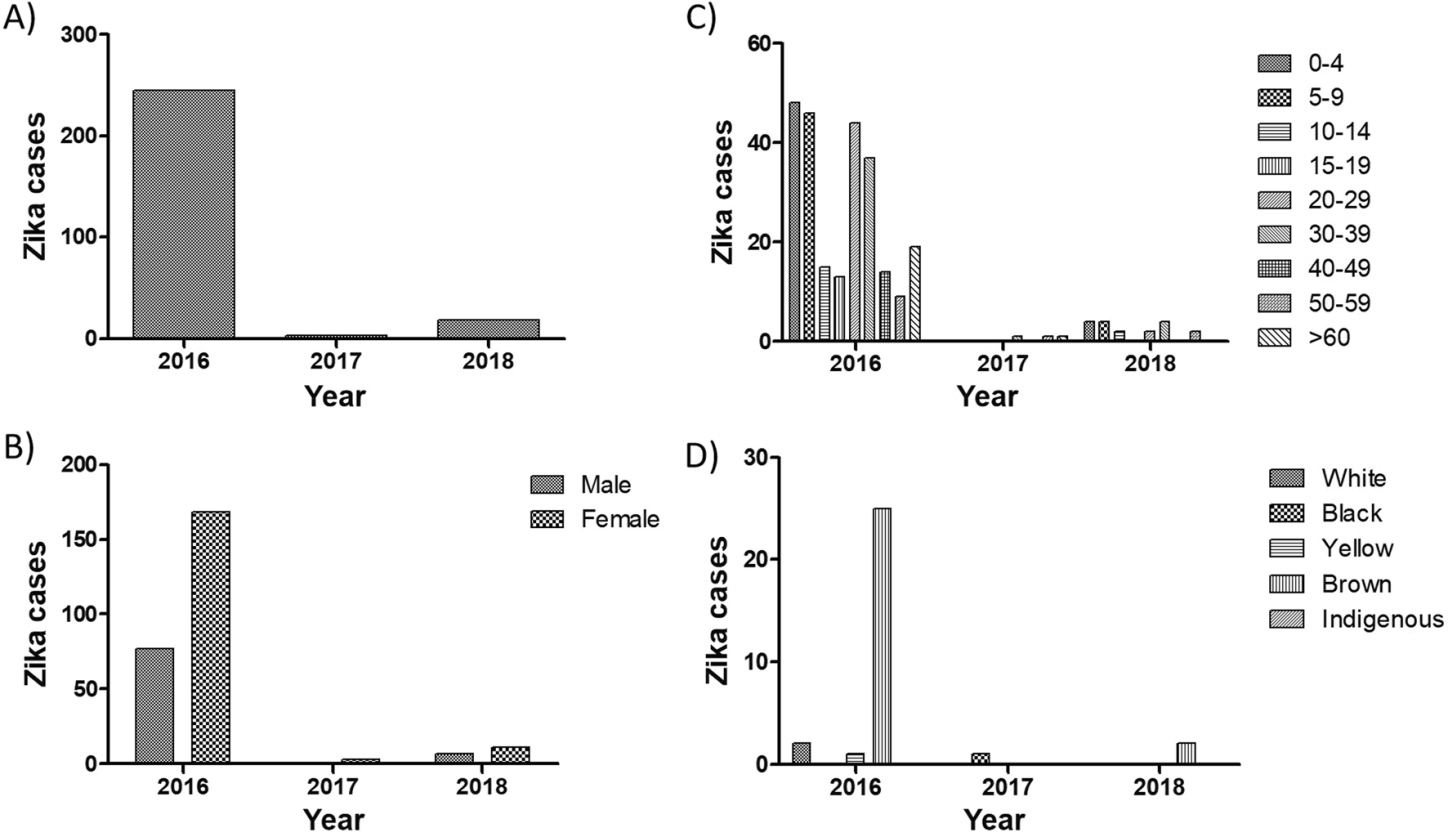
Zika cases in Recife city, Brazil. Zika cases were analyzed considering the total number of cases per year (**A**) and according to sex (**B**), age group (**C**) and race/color (**D**). Data were made available by the Pernambuco Health Department

In 2016, 2017 and 2018, 245, 3, and 18 individuals were diagnosed with Zika, respectively, with a total of 266 cases, most of them female (Fig. 3A). In 2016 and 2018, the female/male ratio was 2.18 and 1.6, respectively (Fig. 3B). The 0–4, 5–9, 20–29 and 30–39 age groups correspond to those with the most Zika diagnoses (Fig. 3C). In general, brown people were the most diagnosed with Zika between 2016 and 2018 (Fig. 3D). Race/color information was not collected from 88.6% (217/245), 66.6% (2/3), and 88.8% (16/18) in 2016, 2017 and 2018, respectively.

### DENV, CHIKV and ZIKV co-infection

Between 2016 and 2018, a total of 119 co-infections were identified: 117 DENV-CHIKV, 1 CHIKV-ZIKV and 1 DENV-CHIKV-ZIKV. Co-infections were mainly described in 2016 (107 cases), followed by 2018 (7) and 2017 (5).

### DENV: 2011–2013 *vs*. 2016–2018

Females were the most diagnosed with dengue in the two triennia (Fig. 1B). However, the female/male ratio decreased significantly between 2011–2013 *vs*. 2016–2018: 1.42 *vs*. 1.3 (*p* < 0.001). This reduction was also observed between the individual years of each triennium: 2011 *vs*. 2018 (1.37 *vs*. 1.06, *p* = 0.0002), 2012 *vs*. 2016 (1.52 *vs*. 1.32, *p* < 0.0001), 2012 *vs*. 2017 (1.52 *vs*. 1.29, *p* = 0.0015) and 2012 *vs*. 2018 (1.52 *vs*. 1.07, *p* < 0.0001) (Fig. 1B). An increase in the female/male ratio was observed only between 2013 *vs*. 2016 (1.07 *vs.* 1.32, *p* < 0.0001) (Fig. 1B).

Regarding age groups, individuals aged 20 to 29 years were the group with the highest number of dengue diagnoses in the two trienniums (Fig. 1C). Browns were the majority of those diagnosed with dengue in all years of study (Fig. 1D), however the frequency increased between 2011–2013 *vs*. 2016–2018 (70 *vs*. 77.4%).

### Climate analysis

**Fig. 4:**
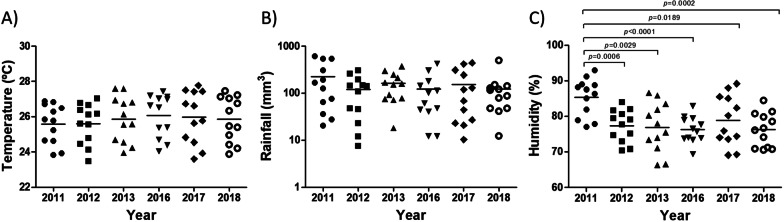
Climate analysis of Recife city, Brazil. Monthly temperature, rainfall and humidity data were analyzed between 2011 and 2013 (DENV circulation) and between 2016 and 2018 (DENV, CHIKV and ZIKV co-circulation). Statistical analyses were performed using the one-way analysis of variance (one-way ANOVA) and paired t-test (*p-value* < 0.05 and 95% confidence interval). Temperature and humidity information was collected from the National Institute of Meteorology (*Instituto Nacional de Meteorologia*, INMET). Rainfall data were obtained from the State Agency of Water and Climate (*Agência Pernambucana de Águas e Clima*, APAC). DENV: dengue virus; CHIKV: chikungunya virus; ZIKV: Zika virus

No significant difference was observed among the temperature averages of the years analyzed: 2011 (25.58 ± 1.11 °C), 2012 (25.6 ± 1.14 °C), 2013 (25.86 ± 1.3 °C), 2016 (26.07 ± 1.21 °C), 2017 (25.98 ± 1.48 °C) and 2018 (25.85 ± 1.3 °C) (Fig. 4A). Similarly, no significant difference was observed in the average rainfall: 2011 (145.2 ± 369.55 mm^3^), 2012 (125.57 ± 137.41 mm^3^), 2013 (161.92 ± 143.71 mm^3^), 2016 (82.02 ± 118.80 mm^3^), 2017 (94.25 ± 252.08 mm^3^) and 2018 (101.25 ± 86.40 mm^3^) (Fig. 4B). Only the humidity of 2011 (85.36 ± 5.32%) showed a significant difference compared to the other years: 2012 (77.36 ± 4.51%; *p* = 0.0006), 2013 (76.9 ± 6.95%; *p* = 0.0029), 2016 (76.3 ± 3.7%; *p* < 0.0001), 2017 (78.88 ± 7.08%; *p* = 0.0189) and 2018 (76.24 ± 4.79%; *p* = 0.0002) (Fig. 4C).

## Discussion

### DENV circulation (2011–2013)

During the last triennium of DENV-only circulation in Recife, Brazil, we observed a high number of dengue cases between 2011 and 2012 and a considerable reduction in 2013. Although the climate may influence the vector population and, consequently, the dengue numbers [11, 13, 14], only humidity in 2011 was significantly different among the analyzed years. Another factor potentially related to the fluctuation of dengue cases is the (re)introduction and/or predominance of different serotypes and/or viral genotypes [7, 8, 34]. The four DENV serotypes circulated in different Brazilian regions during 2011–2013 [10, 35, 36], however, to the best of our knowledge, there is no description of which serotypes circulated in Recife during this period. Despite this, it is important to consider that 2011 was the probable year of introduction of DENV-4 in Pernambuco state [10], which may have contributed to the cases of 2011 and 2012. Unfortunately, the lack of surveillance on DENV serotypes (and/or genotypes) makes it difficult to discuss changes in dengue numbers from a herd immunity or ADE perspective.

Regarding the epidemiological profile, females had more dengue diagnoses than males. Interestingly, the female/male ratio fluctuated according to the total number of cases. According to the latest report by the Brazilian Institute of Geography and Statistics (*Instituto Brasileiro de Geografia e Estatística*, IBGE), the female/male ratio in the general population of Recife is about 1.16 [37], a different value from that observed in our study: 1.37 (2011), 1.52 (2012) and 1.07 (2013). This disagreement suggests that the population profile was not the only factor responsible for our finding. The high number of dengue diagnoses in women was also observed in other years in Pernambuco state [38]. On the other hand, studies carried out in other Brazilian regions found a higher prevalence in men, including a statistically supported association [39, 40].

Individuals aged 20–29 years were the most diagnosed between 2011 and 2013. Although this age group represents most of the population of Recife (18.3%) [37], its percentage is lower than that observed in our study (20.73, 22.74 and 25.60% in 2011, 2012 and 2013, respectively). Like what was discussed above, despite the influence of the population profile on the numbers observed here, possibly this is not the only determining factor for our results. In general, different age groups have been described as the most related to dengue, *e*.*g*., 15–49, 20–29, 21–30, 21–40, 30–39, 31–40, 18 years, ≥ 30 years or > 50 years [15, 17, 38, 41–44]. These disagreements are probably influenced by the region/territory analyzed, as well as by the study design.

Particularly in Brazil, in a retrospective study carried out with data from 2007 to 2017, Einlof et al. [45] described that the age group of 20–29 years was the most frequent in women diagnosed with dengue, especially in pregnant women. Taken together, these data suggest higher vulnerability in this age group, probably related to behavioral and environmental factors. It is possible, for example, that individuals aged 20–29 years, probably more socially active, are more exposed to the vector, presenting a higher risk of infection.

Regarding race/color, brown was the most diagnosed in 2011 (69.6%), 2012 (69.7%) and 2013 (74.2%). The highest prevalence in browns has also been described in Pernambuco state [38]. According to the IBGE, in 2012, 46.3% of Brazilians declared themselves as white, 45.6% as brown and 7.4% as black [46]. Although the racial profile from Recife may differ from that of Brazil, we do not believe that the findings observed here may be explained only by the population characteristics.

In conclusion, we observed that in the last three years of DENV-only circulation in Recife, most dengue diagnoses were reported in females, individuals aged 20–29 years and brown people. Due to the difference between our results and the general population, it is possible to infer that this epidemiological profile may be somehow related to dengue cases/diagnosis.

### DENV, CHIKV and ZIKV co-circulation (2016–2018)

To contribute to the discussions on the influence of CHIKV and ZIKV on dengue epidemiology, we compared the epidemiological profile of dengue cases in the last years of DENV-only circulation *vs*. that observed in the first years of DENV, CHIKV and ZIKV co-circulation. It is important to note that 2014 and 2015 were not included because they were the transition period between DENV and DENV-CHIKV (2014) [29], and between DENV-CHIKV and DENV-CHIKV-ZIKV (2015) [30].

In general, 2016 was the year with the most dengue diagnoses during 2011–2013 and 2016–2018. Interestingly, in 2017 and 2018, few years after the first autochthonous case of chikungunya and Zika in Brazil, we observed a considerable decrease in dengue diagnoses in Recife. This sharp drop was also observed in general data for Brazil. In 2016, a federal law was enacted in Brazil to improve public health surveillance for DENV, CHIKV and ZIKV [47], which may have contributed to the decrease in cases in the following years. However, in the same period, there was a decrease in dengue cases in other American countries, including Central America, Andean subregion, Southern Cone and Latin Caribbean [18, 31, 48], which suggests that this scenario may also be related to changes in the virus-host interaction. Although this issue has not been fully clarified, others studies have discussed some factors, such as potential cross-immunity between DENV and ZIKV [26, 48, 49].

Furthermore, diagnoses of chikungunya and Zika also decreased in Recife during 2017 and 2018. A similar context was observed in Brazil, especially in 2018 [31]. The combined decrease of the three arboviruses leads us to suggest that, in addition to factors related to each virus, such as herd immunity and/or cross-protection, there may be a common cause, *e*.*g*., a reduction in the vector population. This issue was well discussed by Perez et al. [48]. As described for 2011–2013, we also did not find climatic changes that could explain the difference between 2016 and 2018. This constancy of climatic variables is explained by the location of Recife in the tropical region of Brazil.

Importantly, even with the DENV, CHIKV and ZIKV co-circulation, the epidemiological profile in 2016–2018 was similar to that of 2011–2013: female, age group 20–29 years and brown continued to be the most described with dengue. As reported for the DENV-only circulation period, the decrease in the female/male ratio in 2016–2018 is in line with the decrease in total diagnoses.

In addition to analyzing dengue cases, we identified the epidemiological profile of chikungunya and Zika in their early years. Regarding chikungunya, most diagnoses were in females, in a female/male ratio considerably higher than that observed in dengue. The higher diagnosis in women may be related to the higher demand for medical care, since the female sex has been reported as a risk factor for severe joint pain in chikungunya disease [50].

The distribution of chikungunya by age also differed from that found for dengue: while dengue showed a graph with an almost concave shape, with the highest number in 20–29 years, we observed two main age groups for chikungunya; 0–19 years (the minority of diagnoses), and older than 20 years, which covers most diagnoses, including individuals over 60 years old. Considering that individuals with dengue or chikungunya correspond to the same target population, it is possible to suggest that the age difference reflects the epidemiological profile of each arbovirus. A likely reason for the higher diagnosis of chikungunya in individuals over 20 years old would be the higher frequency (and/or severity) of acute illness, which could result in a higher diagnostic demand. Although this hypothesis seems reasonable, we did not find studies that describe adulthood as a risk factor for chikungunya disease. On the other hand, clinical manifestations often associated with acute illness seem to be less common in individuals over 60 years old; this group, however, has been more related to atypical and severe forms and deaths by chikungunya [51, 52]. Regarding race/color, similar to dengue, most individuals diagnosed with chikungunya were browns.

Concerning Zika, females also corresponded to the most described sex. However, the distribution of Zika by age differed from that observed in both dengue and chikungunya. For Zika, the age groups of 0–9 and 20–39 years corresponded to the most diagnosed. This is an important issue and deserves further investigation, especially when considering the risk of congenital Zika syndrome and the fact that the age group of 20–39 years (childbearing age) is among the most diagnosed in our study. Despite this, some authors reported difficulties in carrying out a more in-depth analysis in this population due to the lack of data on pregnancy during the notification of arboviruses [45].

Similar to the one discussed for dengue *vs*. chikungunya: What could explain the difference in diagnosis among arboviruses? Santos et al. [53] reported the highest number of Zika diagnoses in children younger than 4 years. The authors hypothesize that at this age children are closer to women or caregivers in daycare centers or preschools, in environments where the mosquito vector is ubiquitous, contributing to the risk of infection. However, considering that the target population was the same for DENV and CHIKV and that this profile was observed only in ZIKV, we do not believe that this context explains our results. On the other hand, this difference may be partially explained by the greater demand for Zika diagnosis due to the probability of congenital Zika syndrome, although other environmental and social factors may be related.

Our analysis of Zika cases was greatly hampered by the low collection of data on race/color. Moreover, the few diagnoses in 2017 and 2018 also made a more detailed analysis difficult. In addition to the factors related to potential herd immunity, the low number of Zika diagnoses is likely related to the characteristics of the infection, in which about 80% of cases are asymptomatic [54, 55], reducing the probability of diagnosis.

Finally, we identified DENV-CHIKV, CHIKV-ZIKV and DENV-CHIKV-ZIKV co-infections, mainly in 2016, which may be explained by the higher viral circulation in this year. These results are in line with previous studies from Pernambuco, which describe co-infections by these arboviruses [56–58]. Overall, considering that co-infections may lead to aggravation of the disease [56], we believe it is opportune to investigate possible arbovirus co-infections, at least in situations with high clinical-epidemiological suspicion.

## Conclusion

The introduction of CHIKV and ZIKV did not change the epidemiological profile of dengue cases in our study population. Although dengue, chikungunya and Zika showed some epidemiological differences, mainly concerning age, women were the most diagnosed by the three arboviruses in all years analyzed. This finding highlights the need for higher attention regarding prevention and care measures for this population. Finally, considering that our findings were obtained from the epidemiological context from Recife, Brazil, we encourage future studies to analyze the influence of CHIKV and ZIKV co-circulation on dengue cases in other countries/territories, in order to identify local epidemiological profiles, as well as those more consistent in arbovirus dynamics.

### Study limitation

It is important to consider that our study was carried out with diagnostic numbers provided by the Pernambuco Health Department and not with DENV, CHIKV or ZIKV infection numbers. Although this issue is a limitation of many epidemiological studies, we believe that this context must be viewed with care, especially for Zika, since most infected people are asymptomatic and, consequently, do not seek diagnostic services. In addition, race/color was ignored during the diagnosis of most individuals. The lack of such data makes a detailed analysis of the distribution and dynamics of arboviruses difficult.

## Data Availability

The data that support the study findings may be available upon reasonable request to the corresponding author.
